# Twenty-Four-Hour Feeding Patterns of In-Home Healthy Aging Cats Fed Wet, Dry, or a Combination of Wet and Dry Diets Ad Libitum

**DOI:** 10.3390/ani16010045

**Published:** 2025-12-24

**Authors:** Ryan Eyre, Emily Marshall, Annabelle Goyon, Zack Ellerby, Laura Carvell-Miller, Scott J. McGrane

**Affiliations:** 1 Royal Canin Pet Health Nutrition Center, 6574 St. Rt. 503 N, Lewisburg, OH 45338, USA; ryan.eyre@royalcanin.com; 2 Waltham Petcare Science Institute, Freeby Lane, Waltham-on-the-Wolds, Melton Mowbray, Leicestershire LE14 4RT, UK; emily.marshall@effem.com (E.M.); zack.ellerby@effem.com (Z.E.); laura.carvell-miller@effem.com (L.C.-M.); 3 Royal Canin Research Center, 650 Avenue de la Petite Camargue, 30470 Aimargues, France; annabelle.goyon@royalcanin.com

**Keywords:** cat, feeding pattern, dietary format, senior, aging

## Abstract

Domesticated cats have evolved from the obligate carnivore *Felis silvestris*. The instinct to eat small frequent meals and a crepuscular feeding rhythm was conserved during domestication. Declining senses, cognitive impairment, and poor oral health decrease food intake in aging cats. Combined with metabolic and physiological changes, they are at risk of sarcopenia and general body condition impairment. Aging cats may, therefore, benefit from modified nutritional strategies to achieve their metabolic energy requirements; however, little is known about feeding patterns in aging cats and influence of food format. This in-home study of 134 aging cats (≥7 years) assessed ad libitum dry, wet, and wet/dry (‘mixed’) feeding regimens to compare 24 h feeding frequency and caloric intakes. Feeding frequency was significantly different between wet and dry (*p* = 0.02) and mixed and dry diets (*p* < 0.001), with cats consuming 6.0 (dry), 6.9 (wet), or 7.2 meals (mixed) per day. Mean caloric intakes were significantly higher for dry (262.6 kcal/d) and mixed (222.6 kcal/d) regimens (both *p* < 0.001) versus wet (138.1 kcal/d). In conclusion, healthy aging cats eat small frequent meals, with feeding frequency and caloric intake influenced by dietary format. These findings support development of evidence-based feeding guidelines for aging cats.

## Simple Summary

Pet cats have evolved from the African wildcat, inheriting many of their feeding habits. Studies have shown pet cats eat small frequent meals with feeding peaks around dawn and dusk. Like aging humans, aging cats may eat less and need encouragement to eat enough to maintain their body condition and general health. Little is known about how aging affects daily meal frequency and meal size in cats. Knowledge is also lacking on how feeding wet, dry, or a combination of both wet and dry diets (‘mixed’) affects feeding frequency and daily energy intakes of older cats. A total of 134 in-home healthy aging cats aged 7+ years were given free access to a dry, wet, and mixed diet for 24 h a day using automated weighing feeders. Feeding data for each diet were collected for two days; all cats tested all diets in a randomly allocated predefined order. Cats consumed around 6 (dry) or 7 (wet and mixed) small meals over 24 h, clustered around dusk and dawn. They consumed more calories when fed all-dry and fewest calories overall in an all-wet regimen. The study outputs contribute towards development of scientifically supported feeding guidelines for aging cats.

## 1. Introduction

Domesticated cats (*Felis catus*) have evolved from the African wildcat (*Felis silvestris*), an opportunistic solitary hunter. This obligate carnivore hunts and consumes several small items of prey throughout a 24 h period, ensuring a ‘little and often’ intake of food. There is also a physiological reason for this behavior: in comparison with dogs, the cat has a relatively restricted stomach volume of around 300–350 mL, necessitating frequent small meals [1]. Domesticated cats do not generally need to hunt for food when their feeding needs are met by humans. That said, the instinct can compel some house cats to hunt and consume prey several times daily, whether living in urban or rural environments [2]. The inherent drive to eat several small meals throughout the day has been maintained during domestication, with approximately 12–20 feeding occasions of 5–7 g of food reported over a 24 h period [3,4]. This pattern of small frequent daily feeds has been confirmed more recently in domesticated colony cats, showing feeding peaks at dusk and dawn, reflecting the crepuscular nature of this species [5,6,7]. A recent consensus statement recommends providing cats with multiple small meals throughout the day to allow them to display normal feline feeding behavior and to promote wellbeing [8]. By contrast, there is evidence to suggest that feeding cats once per day promotes higher levels of satiety hormones and may help maintain lean body mass compared with feeding four daily meals [9].

Aging cats experience metabolic and physiological changes, which may include a declining ability to digest certain nutrients and impaired protein synthesis, although there is conflicting evidence for this effect [10,11]. The Feline Lifestage Guidelines published by the American Animal Hospital Association (AAHA) and American Association of Feline Practitioners (AAFP) define cats as mature from 7 to 10 years and senior at >10 years [12]. However, the definitions of these stages can vary according to the breed and genetic predispositions [13]. Advances in veterinary care and specialized nutrition are contributing to an increasing lifespan of pet cats, living on average for 12–14 years, and potentially into their 20s [14,15]. These advances may also contribute towards a gradual shifting in the age ranges that define cat life stages. When cat life stages are defined using disease diagnosis data and compared against previous published data, the ages at which a cat is considered mature or senior appear to be later than previously described [16]. In this analysis, late midlife was defined in cats as 10–11 years, senior as 12–13 years, and a further life stage termed supersenior as ≥14 years [16]. For the purposes of the current study, aging cats are defined as ≥7 years, with sub-categories of 7–11 years and ≥12 years. We deliberately chose to study cats from the age of 7 years to capture the point at which they start to be considered mature and begin to show changes, according to the AAHA and AAFP definitions. There is general agreement that from around 12 years of age, cats are entering the senior or geriatric stage, hence our choice of age sub-category thresholds.

As humans age, qualitative and quantitative changes in chemosensory flavor perception can reduce food intake [17]. There is evidence to suggest that aging cats may also display reduced food intake; causal factors include a declining sense of taste and smell, difficulty chewing, and cognitive impairment [13,18]. This decrease in food intake may necessitate modified feeding strategies to encourage sufficient intake of nutrients and energy for maintenance of body condition. Despite acknowledging that aging cats require tailored nutrition to address their expected age-related health changes, the European Pet Food Industry Federation (FEDIAF) does not publish distinct nutritional guidelines for this life stage. In terms of understanding feeding patterns in the aging cat, a small study in a research colony setting observed no statistical differences between natural feeding patterns of adults and seniors [19]. However, this has not since been confirmed in a larger study or in an in-home setting. In the absence of definitive evidence for the ideal feeding frequency of aging cats, providing three or four small meals each day is suggested by the Senior Care Guidelines of the American Association of Feline Practitioners [20]. The main objective of the current study is to examine the 24 h feeding frequency and meal size in aging cats (≥7 years). We hypothesize frequent feeding of small meals in aging cats, as reported previously in adults. The study also aims to understand the influence of dietary regimens (wet, dry, or a wet/dry combination) on the outcomes, hypothesizing a similar feeding frequency of all three regimens. Secondary objectives examine caloric and water intake across the three tested dietary regimens. We expect the all-wet feeding regimen to result in the lowest caloric intake and the highest total water intake, in line with previous literature.

## 2. Materials and Methods

### 2.1. Study Design

This in-home study exposed aging cats to three dietary regimens (dry diet only, wet diet only, and a combination of wet and dry diet) in a randomized, blinded, and order-balanced crossover design. In this design, each animal acted as their own control to mitigate individual differences, such as weight, that might otherwise represent major confounders. The feeding of each dietary regimen comprised a four-day adaptation feeding period, followed by a two-day feeding exposure with data collection and a rest day during which no data were collected. Note that the rest day was introduced to enable three Monday to Saturday test periods for owner convenience. The study was run in the UK from 11 November 2019 to 9 December 2019.

### 2.2. Statistical Power Analysis

Power analyses were carried out *a priori* for both meal frequency and caloric intake, at the respective effect sizes of interest (1 meal and 20 kcal per day). For robustness, the power was estimated by simulation for two separate scenarios, using data from two previous studies involving ad libitum feeding of dry and wet diets, respectively. We report power estimates for the minimum of these two scenarios below, in a conservative approach.

The results indicated that, under a two-exposure design, our initial target sample of n = 150 animals would provide power of >99% for meal frequency and >94% for caloric intake. Our reduced sample following drop-outs, of n = 135 animals, would provide a power of >81% for meal frequency and >91% for caloric intake. For the n = 126 animals providing full datasets, the power would be >63% for meal frequency and >89% for caloric intake. Note that our final effective sample size fell between n = 126 and n = 134, due to how linear mixed-modeling handles animals with incomplete data.

### 2.3. Participating Animals and Inclusion Criteria

In total, 150 senior pet cats aged ≥7 years were recruited to the study. Households could participate in the study if they were willing for their cat to try a new brand of cat food, were connected to broadband with access to their router, had access to mobile phone or tablet applications, and owned up to two cats. Note that although two-cat households were permitted to participate, all the participating cats were from single-cat households. Owners were asked to confirm if they would be at home each day (i.e., not planning any significant time away) for the duration of the study. Cats were excluded if they were perceived by their owner to be either greedy or fussy eaters, not comfortable wearing a collar, timid, or not able to acclimate well to change. Exclusions were also applied if owners noted their animal was displaying increased food consumption due to a medical condition or medication, had a clinically diagnosed condition for which they were either receiving treatment or was related to oral health or tooth issues, were on a special diet, were pregnant or lactating, or had never been exposed to a dry or wet commercial diet. All cats were indoor cats or those with limited outdoor access. Limited outdoor access was defined as outdoor access restricted to the immediate garden area and was owner-supervised to minimize the risk of consumption of alternative food and water sources. During selection, owners were provided information on the study requirements and asked to confirm that they were willing to spend time acclimatizing their cat to use an automated feeder. They also needed to commit to attending a 20 min training session the week prior to the study start date and were asked to confirm their commitment to a total participation period of 36 days and agree to completing questionnaires after each dietary exposure. A cash incentive of 110 GBP was offered on completion of the study.

In this study, 150 cats were randomly assigned to one of the six condition-order groups, without replacement, according to a balanced Latin square design (Table 1). This ensured that comparable numbers of cats were exposed to the feeding regimens in each order, by both position in sequence and preceding and subsequent regimens, to mitigate potential time or carry-over effects (e.g., weight gain over the ad libitum study period).

Due to acclimation issues and/or personal reasons, 15 cats were lost from the study prior to the first data collection phase. Of the 135 cats that completed the study, one cat was removed from the data set due to repeated highly irregular data variables to give a final analysis with 134 cats. The majority of the 134 analyzed cats were domestic shorthair (n = 117); the remaining cats were domestic longhair (n = 14), mixed breed (n = 2), and other (n = 1). There were 70 cats in the 7–11 years of age category (34 males, 36 females, mean age of 8.7 years, range: 7–11 years) and 64 cats in the ≥12 years of age category (26 males, 38 females, mean age 13.8 years range: 12–22 years). Note that age data were not submitted by owners for all cats; therefore, the mean ages and ranges are based on 42 cats in the 7–11 years group and 39 cats in the 12+ years group.

### 2.4. Ethics Statement and Data Handling

This study conformed with the Mars Animal Research Policy (www.mars.com, accessed on 1 October 2025) and was approved by the Waltham Animal Welfare and Ethical Review Body (AWERB), project number 75987. It also followed the 3Rs approach to experimentation with animals in scientific research [21] and was conducted in accordance with the local guidelines and regulations. Reporting of the animal studies conforms to the ARRIVE guidelines [22]. All owner and pet data adhered to the General Data Protection Regulation (GDPR) and Market Research Society (MRS) codes of conduct.

### 2.5. Feeding Apparatus and Acclimation

**Figure 1:**
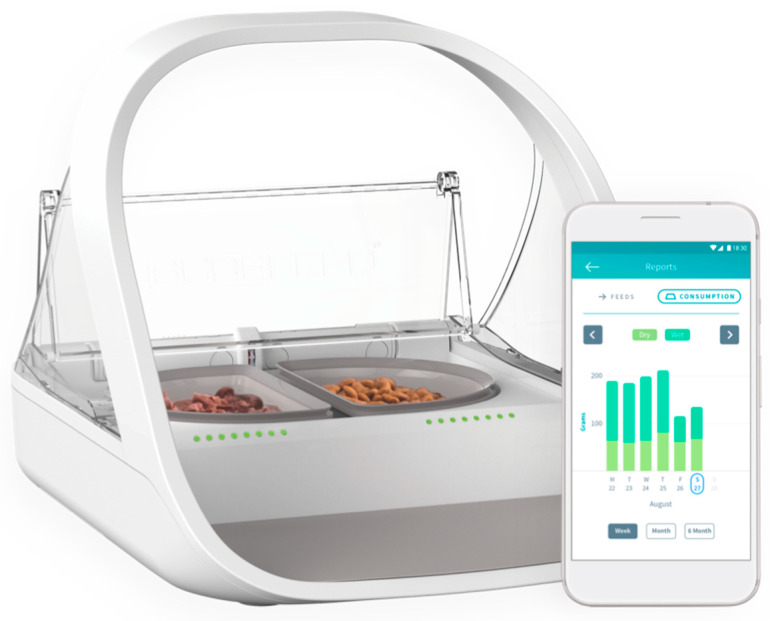
The Sure Petcare Microchip Pet Feeder Connect used in the study. Taken from www.surepetcare.com/en-gb/pet-feeder/microchip-pet-feeder-connect, accessed on 1 October 2025.

The Sure Petcare Microchip Pet Feeder Connect feeding station (model IMPF; Sure Petcare, MSD Animal Health, manufactured in China) containing two built-in scales was used to feed the cats (Figure 1). Each feeder was operated by a unique Radio Frequency Identification (RFID) collar tag on each cat, allowing exclusive use of the feeder by an individual cat. Although the feeder can also be operated via the cat’s microchip implant, use of the RFID collar enabled the full blinding of the study. The feeding system allowed pet owners to provide either one diet in a single bowl, or, in the case of the mixed wet/dry regimen, two diets simultaneously in separate bowls. An automated lid which closes between feedings maximized diet freshness throughout the day. However, any wet food remaining after approximately 8 h was replaced with freshly opened diet. Data from the feeding system were available in the Sure Petcare mobile application via the Sure Petcare Hub (model IHB), connected to the home router.

Owners collected the automated feeder at a training session during which they were shown how to operate the feeder and any questions were answered. Prior to the study start, cats spent 14 days acclimating to the feeding system using their usual diet and were acclimated to ad libitum feeding for the final four days of this period prior to entering the test phase. The acclimation period was an opportunity to note any changes in the cats’ feeding behavior as a result of introducing the automated feeder and to identify cats that could not adapt to the apparatus. During this period, and throughout the test phase, owners had access to live technical support to mitigate any technical issues with the feeding apparatus.

### 2.6. Diets and Feeding

Cats aged 7–11 years were offered Royal Canin^®^ Instinctive 7+ chunks in gravy (CIG; wet), Royal Canin^®^ Indoor 7+ (dry), and a combination of both diets. Cats aged ≥ 12 years were offered Royal Canin^®^ Ageing 12+ CIG (wet), Royal Canin^®^ Ageing 12+ (dry), and a combination of both diets (see Table 2 for dietary analysis information and Table S1 for full ingredients lists of the test diets). During the test phase, at the beginning of each day, cats were offered approximately 200–250 g of wet diet (replaced with a freshly opened product after approximately 8 h) for the wet feeding regimen, approximately 200 g of dry diet for the dry feeding regimen, or 85 g of wet and 100 g of dry (in separate bowls) for the combination feeding regimen. The diets were replenished as necessary to maintain free access to food. This protocol enabled the diurnal and nocturnal free-feeding of the cats over a 24 h period in which they chose when to feed and how much.

All cats were offered water ad libitum separate from the feeding stations. To minimize evaporation losses, the water was replenished each time the wet food was replaced or approximately every eight hours when feeding only a dry diet. The owners weighed the water containers at the start and end of each replacement, using a provided simple electronic balance (ABN Finest, 5 kg limit) to generate data on water consumption.

### 2.7. Data Collected

The owners reported their cat’s gender and age category (7–11 years or ≥12 years). A subjective owner-assessed initial body condition score (BCS) was also submitted (see Supplementary Table S2). For each of the three feeding regimens, feeding data were captured for two 24 h exposure periods for each cat. Although in-home studies often collect single exposure data for each diet, dual exposure designs offer higher statistical power. The power analyses indicated that the assessment of both intake and meal frequency to our desired precision would require two exposures per cat, for an economically viable number of animals and equipment. Data were also collected during the four-day adaptation phases to provide additional feeding data for secondary analysis. Data collected from the automated feeding system were captured on a mobile application which tracked the frequency of feeding (including the time of each feed), total food consumed at each feeding, and duration of each feeding. Data from the mobile application were automatically uploaded to Sure Petcare and were subsequently transferred to the study owners. After the two-day exposure to each regimen, owners were asked to complete an online questionnaire, which gathered information on amount of wet and/or dry food eaten, number of times the cat fed, and time spent at the feeder (see Supplementary Table S3). Water consumption data (voluntary water intake, measured in g) were also gathered for each cat, and this was combined with the calculated dietary moisture intake to give total water intake. Water spillages were not recorded and were assumed to be randomly distributed across groups.

### 2.8. Statistical Analyses

All data were statistically analyzed in R version 4.2.2. [23]. For primary analyses (number of meals and caloric intake across 24 h), only data from days 5 and 6 were used, in line with the powering requirements.

To assess meal frequency, a linear mixed effects model was fitted to the number of meals consumed per day as the response variable, where a single meal was defined as each occasion the cat went to the food bowl and ate at least 2 g of food, with no interprandial pause included. The calories consumed per day were calculated based on the amount of the dry and/or wet food consumed, using dietary calorie density values in Table 2, and modeled as a response variable in another linear mixed effects model. Both models used the same structure, with dietary regimen as a categorical fixed effect, and diet format crossed and potentially correlated with an individual cat as the random structure (i.e., random intercept and diet-slope per cat). Pairwise comparisons were made between each dietary regimen, using the multcomp package function glht, with single-step family-wise error (FWE) adjustment applied within each model [24]. An additional Bonferroni adjustment was made to the alpha-criterion across the two primary analyses.

Order effects were also explored for both primary analyses, through including the study group (i.e., order) in the model. No statistically significant effect of order was found, either for any individual group coefficient or for their combined impact on the model fit, assessed through the likelihood ratio test. The impacts on mean estimates for each feeding regimen were negligible; so, the results of the simpler models are reported for parsimony.

As a secondary analysis, all analyses undertaken for the primary objectives were repeated using data collected on all days (days 1–6). Gender and age category were also investigated for their effect on the number of meals eaten and calorie intake. For the number of meals, a linear mixed effects model was fitted with the number of meals as the response variable, the variable being explored (age category or gender) as a fixed effect, and the individual cat as a random effect. Another linear mixed effects model was fitted with calorie intake as the response variable, the exploratory variable (age category or gender) as a fixed effect, and the diet format crossed with individual cat as the random effect.

Time effects were also explored across the six-day period for both meal frequency and caloric intake, through including the study day in the model as a continuous fixed effect, both independently and in two-way interaction with feeding regimen. No statistically significant effect of day was found, for either main or any interaction coefficient, or for overall model fit assessed through the likelihood ratio test. The impacts on mean estimates for each feeding regimen were also negligible; so, the results of the simpler models are reported.

To analyze the effect of the feeding regimen on water intake, the water intake was fitted in a linear mixed effects model with water consumed (voluntary water intake) as the response variable, the dietary regimen as a fixed effect, and the individual cat as a random effect. A further analysis was conducted taking into consideration the additional water consumed through diet, using dietary moisture values in Table 2, to give the total water intake. The same model was then fitted, and comparisons were made between dietary regimens.

The same analyses were repeated with gender and age category as fixed effects in place of the dietary regimen to assess their effects on the voluntary and total water intake. Comparisons were made between the different gender and age groups.

An exploration into the time of day cats were eating was also carried out by plotting a histogram of meals over time for all cats across all dietary regimens, with a table showing the frequencies of meals during the day and night. Here, night was defined as between 6.30 pm and 6.30 am.

All results are given as mean estimates with lower and upper 95% confidence interval (CI) bounds, applying significance thresholds of *p* = 0.025 for the two primary analyses (Bonferroni adjusted), and *p* = 0.05 for all secondary analyses. Both CIs and *p*-values are simultaneous, i.e., family-wise error (FWE)-adjusted within each model.

## 3. Results

Of the 134 cats that completed the study, eight did not complete at least one of the feeding phases (data were missing for phases 2 and 3 in four cats, phase 2 data were missing for two cats, and phase 3 data were missing for two cats), which provides full datasets from 126 cats.

### 3.1. Frequency of Feeding

**Figure 2:**
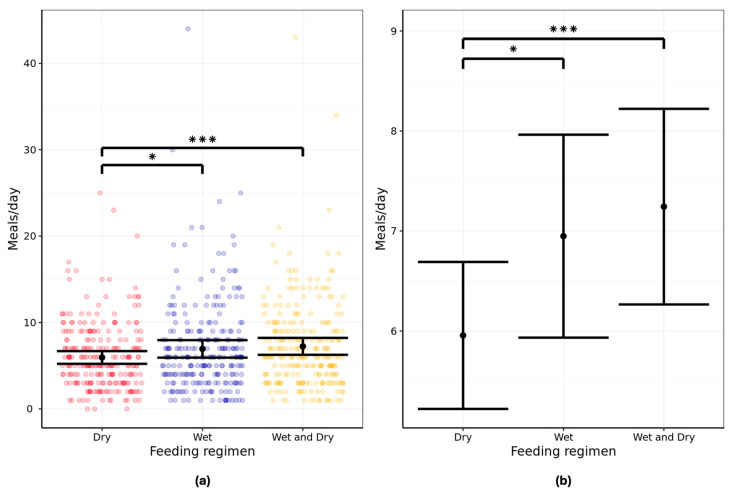
Twenty-four-hour frequency of feeding (meals per day) in pet aging cats (n = 134) fed all-dry, all-wet, or mixed wet/dry regimens showing (**a**) individual data points and (**b**) group means with error bars representing lower and upper 95% CI. Data analyzed from two-day exposure, i.e., last two days of feeding. * denotes difference between regimens is significant at *p* < 0.025. *** denotes difference between regimens is significant at *p* < 0.001.

Analysis of the two-day exposure data from each feeding phase showed cats fed the least frequently in a 24 h period on the all-dry feeding regimen. The mean number of meals consumed for aging cats were 6.0 (95% CI: 5.2–6.7) for the dry regimen, 6.9 (95% CI: 5.9–8.0) for the wet regimen, and 7.2 (95% CI: 6.3–8.2) for the wet/dry regimen per 24 h period (Figure 2; Table S4).

The differences in meal frequency were found to be significant when comparing dry and wet (*p* = 0.02) and dry and mixed wet/dry regimens (*p* < 0.001). However, the difference in feeding frequency between wet and mixed wet/dry was non-significant (*p* = 0.55).

Secondary analysis of the data from all six feeding days resulted in similar means compared with the two-day exposure analysis, with 6.0 (95% CI: 5.3–6.7), 7.1 (95% CI: 6.1–8.0), and 7.3 (95% CI: 6.5–8.2) meals consumed per 24 h period when fed dry, wet, or wet/dry regimens, respectively (Table S4 and Figure S1). As for the primary analysis, the differences between dry and both other regimens were statistically significant (in this case both *p* < 0.001), but between wet and mixed diets they remained non-significant (*p* = 0.4). When the feeding frequency of wet and dry food was examined in the mixed wet/dry feeding regimen, cats consumed a mean of 4.5 meals of dry format (95% CI: 4.0–5.0) and 5.6 meals of wet format (95% CI: 4.9–6.4; Table S2).

### 3.2. Caloric Intake

**Figure 3:**
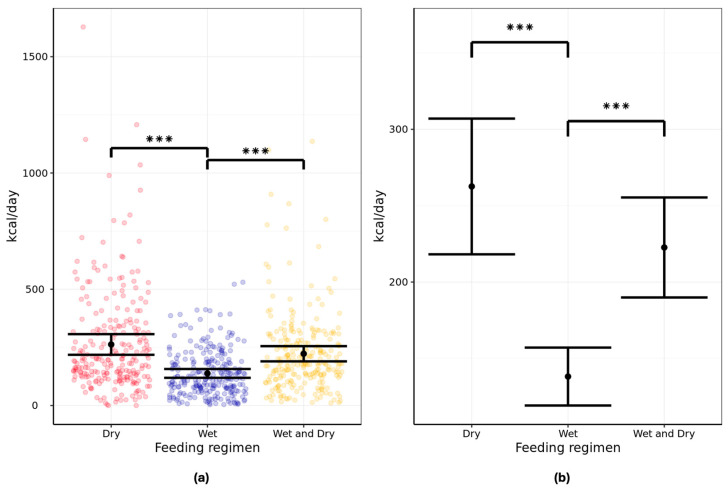
Twenty-four-hour energy intake (kcal/day) in pet aging cats (n = 134) fed all-dry, all-wet, or mixed wet/dry regimens showing (**a**) individual data points and (**b**) group means with error bars representing lower and upper 95% CI. Data analyzed from two-day exposure, i.e., last two days of feeding. *** denotes the difference between regimens is significant at *p* < 0.001.

Analysis of the two-day exposure data from each feeding phase showed differences in the mean 24 h caloric intakes amongst the three dietary regimens. The mean energy consumption was 262.6 kcal/day (95% CI: 218.2–307.0) when fed a dry diet, 138.1 kcal/day (95% CI: 119.2–157.1) when fed a wet diet, and 222.6 kcal/day (95% CI: 189.9–255.4) when fed a wet/dry mix (Figure 3; Table S5). The differences between the wet and dry diets and between wet and wet/dry mix were statistically significant (*p* < 0.001).

The secondary analysis of all six feeding days showed a mean energy consumption of 268.3 kcal/day (95% CI: 227.0–309.6) when fed a dry diet, 148.3 kcal/day (95% CI: 130.2–166.3) when fed a wet diet, and 224.5 kcal/day (95% CI: 197.2–251.8) when fed a wet/dry mix. In this case, the differences between all dietary regimens were statistically significant (Table S5 and Figure S2).

Analysis of caloric intake contributions from wet and dry formats in the wet/dry mix feeding regimen showed that when offered both formats, cats consumed significantly more calories from the dry (263.7 kcal/day) compared with the wet format (182.6 kcal/day; *p* < 0.001; Table S5).

### 3.3. Average Meal Size

Analysis of the six-day feeding periods showed that cats consumed on average 41–43 kcal of dry diet, 19–22 kcal of wet diet, and 29–31 kcal of wet/dry mixed diet per meal. Based on the calorie densities of the diets fed, this correlates to approximately 11 g dry food, 21–24 g wet food, and 17–19 g of the wet/dry mix per meal.

### 3.4. Time of Feeding

**Figure 4:**
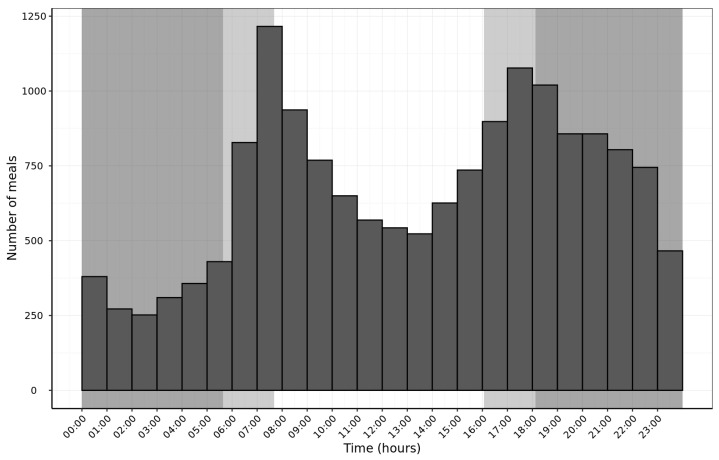
Histogram showing total hourly number of meals consumed for all diets in pet aging cats (n = 134). Data shown are totals summed across the six-day feeding period. Unshaded background indicates period of time between sunrise and sunset, lighter shaded segments indicate astronomical twilight periods (dawn and dusk), and darker shaded segments indicate night-time. All times are calculated as averages over the study time period, for Leicester, UK.

The hourly analysis of food intake for all diets across the six-day feeding period showed two distinct feeding peaks at 06:00–09:00 h and 16:00–19:00 h. Overall, the aging in-home cats consumed food mostly between the hours of 06:30–18:30 and showed decreased feeding in the middle of the night and middle of the day (Figure 4).

### 3.5. Water Intake

#### 3.5.1. Voluntary Water Intake

**Figure 5:**
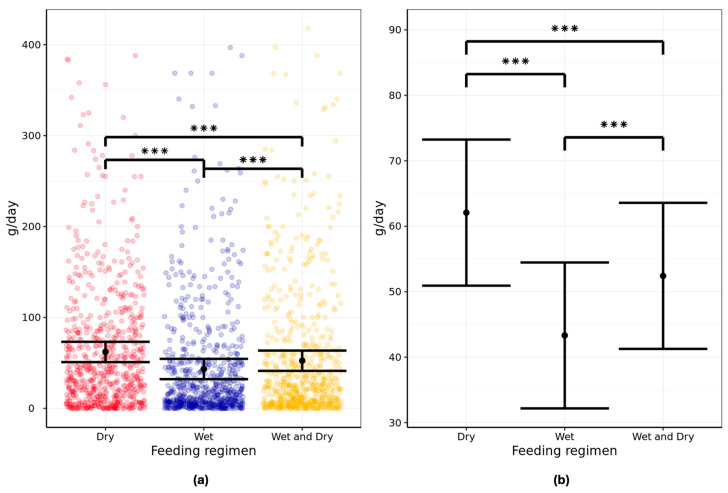
Twenty-four-hour voluntary water intake (g) in pet aging cats (n = 134) fed all-dry, all-wet, or mixed wet/dry regimens showing (**a**) individual data points and (**b**) group means with error bars representing lower and upper 95% CI. Data analyzed from six-day feeding period. *** denotes difference between regimens is significant at *p* < 0.001.

The mean 24 h voluntary water intake (i.e., water drunk) was significantly different between all three dietary regimens (*p* < 0.001), whereby cats drank the most when fed the dry regimen and the least when fed the wet regimen. Cats drank a mean of 62.1 g water (95% CI: 50.9–73.2) when fed the dry regimen, 43.3 g (95% CI: 32.2–54.5) when fed the wet regimen, and 52.4 g (95% CI: 41.3–63.6) when fed the wet/dry mix (Figure 5; Table S6).

#### 3.5.2. Total Water Intake

**Figure 6:**
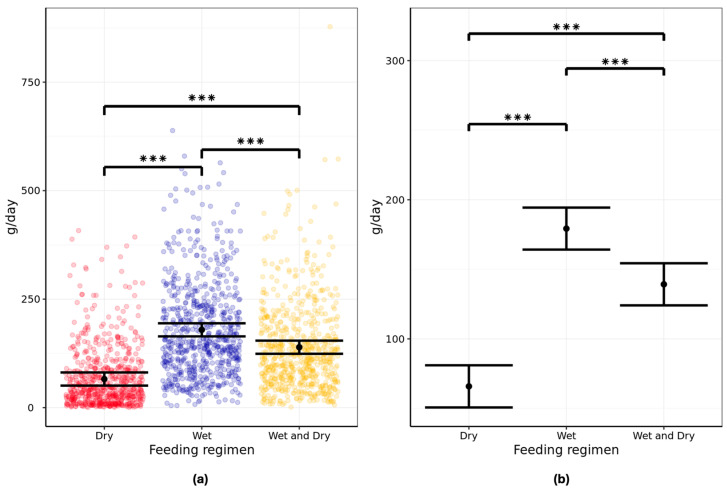
Twenty-four-hour total water intake (water drunk voluntarily plus dietary moisture; g) in pet aging cats (n = 134) fed all-dry, all-wet, or mixed wet/dry regimens showing (**a**) individual data points and (**b**) group means with error bars representing lower and upper 95% CI. Data analyzed from six-day feeding period. *** denotes difference between regimens is significant at *p* < 0.001.

When the dietary moisture intake was combined with the voluntary water intake to give the total water intake, there were again significant differences between all dietary regimens. The mean total water intake was 65.9 g (95% CI: 50.7–81.0) for the dry regimen, 179.3 g (95% CI: 164.2–194.3) for the wet regimen, and 139.2 g (95% CI: 124.1–154.4) for the wet/dry feeding regimen (Figure 6; Table S6).

### 3.6. Influence of Gender and Age Category

There were no statistically significant effects of cat gender or age category on the 24 h feeding frequency in any of the dietary regimens (Tables S7 and S8). In all-diet analyses, the age and gender did not significantly influence the caloric intake, voluntary water intake, or the total water intake (Tables S9 and S10).

## 4. Discussion

This is the first study to examine feeding patterns in pet aging cats in the home environment, using an automated feeding system. The study also collected data to explore the impact of dietary regimen on feeding frequency, calorie consumption, and water intake.

Our findings demonstrate that aging cats fed ad libitum consume on average 6 or 7 small meals throughout a 24 h period, with a statistically reduced feeding frequency on an all-dry regimen compared with all-wet and a wet/dry mix. This frequent feeding of multiple small meals supports our original hypothesis. The biological relevance of the difference between an average of 6 meals per day (dry) and 7 meals per day (wet) on an all-wet regimen is questionable, and the statistical significance is likely a result of the large number of cats participating in this study; therefore, the associated hypothesis is also supported by the data. However, the increased feeding frequency of the wet diet compared to dry makes physiological sense when accounting for the lower calorie density of wet format. The feeding frequency observed here is in approximate agreement with the reported 8 meals consumed across the same timeframe in a study of colony cats fed dry food ad libitum [5]. This study also used automated feeders and defined a meal as one or more eating bouts in which at least 2 g of food was consumed but separated by an interprandial pause of at least 20 min. In contrast, an older study reported a mean of 16 meals consumed per day in laboratory cats fed wet or dry food, with similar feeding frequencies reported for each format [3]. The reasons for this discrepancy lie largely in the study design and environment. This study did not clearly define a meal, and some meals were less than 2 g. Cats were kept in metabolism cages, and this restricted environment may have prompted increased feeding frequency due to fewer distractions and the proximity of food. In addition, the study included only eight cats and was therefore unlikely to be suitably powered. Similarly, a small study (n = 12) of wet diet ad libitum-fed domesticated colony cats reported consumption of 12–14 small meals per 22 h period [19]. Again, direct comparison with the current study is hampered by the lack of definition of a meal, the low numbers of animals, and the different environment. The average meal size in our study ranged from around 20 kcal (wet) to 40 kcal (dry), and this aligns with the approximate 40–50 kcal content of a small mouse, considered a typical prey item [25].

Secondary analysis of the feeding frequency of wet and dry formats within the wet/dry feeding regimen showed cats consumed a mean of 4.5 meals of dry and 5.6 meals of wet format over a 24 h period, seemingly totaling 10.1 daily meals versus the 7.2 wet/dry meals reported in the primary analysis. Whilst this may appear as a discrepancy, this may be explained by a potential overlap, whereby cats consuming the mixed wet/dry regimen fed from both the wet and dry bowls at one sitting.

Our study showed that in a free-feeding situation, aging cats consumed significantly fewer calories on an all-wet dietary regimen compared with a mixed wet/dry or an all-dry regimen, as per our hypothesis. Their calorie consumption was also significantly lower for the wet/dry mixed regimen compared with the all-dry. The recommended daily energy intake for cats is subject to several variables including life stage, activity level, body condition, and neuter status. A 4 kg adult cat with moderate activity levels is estimated to require around 208 kcal per day, according to the following equation: maintenance energy requirement (MER) = 77.6 × BW^0.711^ kcal/day, where BW denotes the body weight in kg [26]. There is no consensus on whether this recommendation should be adjusted for aging cats, as this depends more on activity levels and body condition than age per se. In both aging dogs and humans, it is estimated that energy needs decrease by around 20% due to reductions in activity and the basal metabolic rate [27]. However, some reports suggest that the MER of aging cats should remain the same as adults, or even increase from 12 years of age, due in part to reduced digestive efficiency [27,28]. When fed an all-wet regimen ad libitum, the aging cats studied here consumed a 24 h average of 138 kcal, which falls below the daily recommended MER for a 4 kg cat. Conversely, the mean daily calorie intake of cats fed the all-dry regimen exceeded the recommendations at 263 kcal, whilst the wet/dry mixed regimen resulted in a mean daily energy consumption of 223 kcal. The estimated daily energy requirement of 208 kcal is based on moderate activity levels; the cats studied here were indoor cats or those with minimal outside access, which is likely to reduce their habitual activity levels and daily energy needs. Interestingly, inter-individual variation in the 24 h calorie intake was high for the dry and wet/dry mix regimens and reasonably low for the all-wet. Although the study criteria theoretically excluded cats that were known to be greedy eaters, some opportunistically over-fed when offered dry format to excess, but stomach fill may have naturally limited their intake to a certain extent when fed the all-wet regimen. Overall, the observations indicate that aging cats may not achieve sufficient daily energy intakes when fed wet food solus; so, this may not be an ideal format for older cats prone to sarcopenia and loss of body condition. When considering how this may have affected the health of the cats, a study has shown that the physical welfare of cats was not significantly affected by a 9-month period of calorie restriction; therefore, the very short-term reduction (6 days) in daily calorie intake seen here is unlikely to be of concern [29]. The current study did not collect body weight data throughout the testing period; therefore, it is not possible to determine the actual energy requirements of our studied population or to conclude how the different dietary regimens affected weight maintenance. It must also be noted that the data from the 6-day feeding periods may be insufficient to draw conclusions around adequate energy intakes; studies have shown that cats can take several weeks or months to adapt intakes of low-calorie density diets such that they achieve adequate energy intakes for maintenance [30,31].

The feeding peaks around dawn and dusk observed in our study agree with previous reports [5,6,7]. Our study was conducted in winter in the UK, during which dusk and dawn occur at around 16:00 h and 08:00 h respectively. It has been confirmed that, in domesticated colony cats, these feeding peaks shift slightly across the seasons to coincide with the changing sunrise and sunset hours [7]. However, other factors must be considered that can influence the timing of cats’ meals in an ad libitum fed setting. A strong trigger of food consumption in the cat appears to be human interaction or the presence of a familiar human [7,32]. Cats are known to prefer fresh food in the wild, avoiding consumption of carrion [33]. Pet cats are, to some extent, reliant on humans for their food and can associate human presence with the provision of fresh food, hence triggering a feeding moment. Our study did not standardize this variable, and some owners may have been present all day, whilst others were out at work. There were also no guidelines given regarding interaction with the cats. As this was an ad libitum feeding study, the cat owners were required to maintain a constant supply of food, and in the case of wet food, it was recommended that any food remaining after 8 h was disposed of and replaced. However, there were no stipulated times of day for food replenishment, and this would have revolved around the convenience to the owner. All participating cats were indoor cats or those with limited outdoor access; it is likely that indoor cats are more ‘in tune’ with their owner and are, therefore, more likely to be influenced by their presence, as demonstrated in a study comparing feeding patterns of indoor and outdoor cats [6]. We therefore cannot rule out the possibility that some of the feeding peaks seen in our study may have coincided with human interaction, e.g., a breakfast time food top-up prior to leaving for work and an early evening return from work interaction.

The benefits of the wet format in terms of the water intake must also be considered. The cats studied here drank significantly less water on the all-wet regimen compared with the all-dry regimen. However, the total water consumed was significantly higher for the all-wet versus all-dry regimen, confirming our hypothesis. This observation has been made previously in studies of cats consuming wet or high moisture diets [3,34,35]. The wild ancestor of the domesticated cat obtains most of its daily water requirement from prey, resulting in an inherently low thirst drive and slow responses to changes in hydration. This makes cats especially susceptible to feline lower urinary tract diseases (FLUTD), and this risk appears to increase in older cats [36,37]. Feeding high moisture diets was shown to significantly increase urinary dilution and reduce the risk of calcium oxalate stones in cats [34].

One of the main strengths of this study is the use of automated feeders, incorporating the automatic weighing of food and data capture via an app for accurate and reliable data collection. The added benefit of the feeding system used here was the lid that helped to maintain the freshness of the diet, especially important for the wet format. Whilst it is recognized that a certain level of investment is required in terms of purchasing the automatic feeders and training owners in their use, feedback from owners suggested that the system was relatively simple to use. The strict inclusion criteria for participating households and cats were another strength of the study, minimizing the risk of outlying feeding behaviors and interference from external factors. The large cohort of 134 cats meant the study was powered to detect relatively subtle effects, while increasing the precision of model estimates and confidence in the conclusions. The measurement of water intake via a separately weighed source allows further insights into how aging pet cats voluntarily manage their fluid intake when fed different formats. Finally, the study of pet cats in their home environment is a strength of this study, as the resulting findings are representative of and applicable to the privately-owned cat population.

It is acknowledged that the current study also has some weaknesses. While in-home studies benefit from high ecological validity and can leverage individual owners’ unique knowledge of their own pets, they are naturally hampered by a reduced level of experimental control and the inherently subjective nature of owner perceptions. Pertinent examples include inter-household variation in owner presence, lifestyle, provision of outdoor access (which may have introduced alternative food and water sources), and behavioral schedules (e.g., times of sleep, waking, food replenishment); as well as relying on owner judgements of their cat as greedy or fussy and the accurate reporting of medical conditions. In excluding animals with CKD, diabetes, and clinically diagnosed oral health conditions, we may have limited our study population, though we aimed to standardize to a healthy population as far as possible to minimize confounding influences. The possibility that cats with undiagnosed oral health conditions participated in the study is also acknowledged. The potential effects of food neophobia (reluctance to eat or avoidance of eating new foods) or food neophilia (willingness or tendency to eat new foods) should also be considered in short-term feeding studies such as this. These phenomena have been previously described in cats and are known to impede feeding studies unless managed via a period of prior exposure [38,39]. In this respect, the short two-day primary data collection period is a noteworthy limitation of the current study. However, the consistency of the secondary intake results, observed across the six-day combined adaptation and exposure periods, suggest that our cats were well-acclimated, and neither food neophobia nor neophilia were likely to have substantially impacted the outcomes.

Gaps in the scientific understanding of cat aging processes have been recently acknowledged, as has the lack of definition around what constitutes healthy aging [40]. The current study is an example of the ongoing advancement in understanding changes in feeding behaviors as cats age; this knowledge will ultimately feed into proactive measures to support healthy aging in these pets.

## 5. Conclusions

We have shown that aging pet cats eat 6 or 7 small meals throughout the day with feeding peaks around dusk and dawn and feeding troughs in the middle of the day and night. A significantly reduced mean daily caloric intake was observed when cats were fed an all-wet diet ad libitum compared with all-dry diet. Together with the significantly increased total water intake associated with wet format and its known advantage regarding urinary tract health, the outcomes suggest that aging cats may benefit from a mixture of both wet and dry dietary formats in their daily ration.

The feeding guidelines for aging cats (≥7 years) should recommend feeding several small meals per day using a combination of wet and dry formats to satisfy the aging cat’s grazing behavior, balance calorie intake, and support urinary tract health benefits.

## Figures and Tables

**Table 1:** Order of presentation of feeding regimens, by group.

Group	Feeding Regimen Presentation			Number of Cats
1	Wet	Dry	Wet/Dry	25
2	Dry	Wet/Dry	Wet	25
3	Wet/Dry	Wet	Dry	25
4	Wet	Wet/Dry	Dry	25
5	Dry	Wet	Wet/Dry	25
6	Wet/Dry	Dry	Wet	25

**Table 2:** Analysis of diets used in study, taken from product packaging.

	Royal Canin^®^ Instinctive 7+ Wet	Royal Canin^®^ Ageing 12+ Wet	Royal Canin^®^ Indoor 7+ Dry	Royal Canin^®^ Ageing 12+ Dry
Energy (kcal/kg)	855	935	3776	4080
Fat (%)	2.5	4.0	13.0	19.0
Protein (%)	10.5	9.5	27.0	30.0
TDF (%)	1.5	1.3	12.4	10.9
Moisture (%)	80.5	80.0	6.5	6.5
Ash (%)	1.1	1.2	6.9	5.3
Crude fiber (%)	1.4	1.1	3.6	4.4

All dietary analyses refer to an ‘as fed’ basis.

## Data Availability

The raw data supporting the conclusions of this article will be made available by the authors upon reasonable request.

## Supplementary Materials

The following supporting information can be downloaded at https://www.mdpi.com/article/10.3390/ani16010045/s1, Table S1: Ingredient list of test diets; Table S2: Initial cat body condition scores (BCS) submitted by owners; Table S3: Questionnaire used by owners following each dietary exposure. Table S4: Analysis of feeding frequency (number of meals) in a 24 h period, according to dietary regimen; Table S5: Analysis of caloric intake in a 24 h period, according to dietary regimen; Table S6: Analysis of voluntary water intake (water drunk in g) and total water intake (voluntary plus dietary moisture in g) in a 24 h period, according to dietary regimen; Table S7: Effect of gender on 24 h feeding frequency, by dietary regimen; Table S8: Effect of age category on 24 h feeding frequency, by dietary regimen; Table S9: Effect of gender on 24 h caloric intake, voluntary water intake, and total water intake; Table S10: Effect of age category on 24 h caloric intake, voluntary water intake, and total water intake. Figure S1: Twenty-four-hour frequency of feeding (meals per day) in pet aging cats (n = 134) fed all-dry, all-wet, or mixed wet/dry regimens; Figure S2: Twenty-four-hour energy intake (kcal/day) in pet aging cats (n = 134) fed all-dry, all-wet, or mixed wet/dry regimens.

## Abbreviations

The following abbreviations are used in this manuscript:

AWERB	Animal Welfare Ethical Review Body
BW	Body weight
CI	Confidence interval
CIG	Chunks in gravy
FLUTD	Feline lower urinary tract diseases
FWE	Family-wise error
GDPR	General Data Protection Regulation
MER	Maintenance energy requirement
MRS	Market Research Society
RFID	Radio Frequency Identification
