# Supplementary material for: Twenty-Four-Hour Feeding Patterns of In-Home Healthy Aging Cats Fed Wet, Dry, or a Combination of Wet and Dry Diets Ad Libitum
Source: Animals (Basel). 2025 Dec 24;16(1):45. doi: 10.3390/ani16010045 (PMC12784832; doi:10.3390/ani16010045)
Supplement: Supplementary file 1 [file animals-16-00045-s001.zip › animals-3988315-supplementary.pdf]

Supplementary Information

Table S1. Ingredients lists of test diets.

Diet	Ingredients
**Royal Canin® Instinctive 7+ wet**	Meat and animal derivatives, cereals, vegetable protein extracts, derivatives of vegetable origin, minerals, various sugars, molluscs and crustaceans. Vitamin D3: 320 IU, E1 (Iron): 3 mg, E2 (Iodine): 0.3 mg, E4 (Copper): 2.3 mg, E5 (Manganese): 0.8 mg, E6 (Zinc): 8 mg.
**Royal Canin® Ageing 12+ wet**	Meat and animal derivatives, cereals, vegetable protein extracts, oils and fats, derivatives of vegetable origin, minerals, various sugars, molluscs and crustaceans. Vitamin D3: 300 IU, E1 (Iron): 4 mg, E2 (Iodine): 0.34 mg, E4 (Copper): 2.7 mg, E5 (Manganese): 1.3 mg, E6 (Zinc): 13 mg.
**Royal Canin® Indoor 7+ dry**	Maize, dehydrated poultry protein, barley, maize flour, wheat, vegetable protein isolate*, animal fats, maize gluten, hydrolysed animal proteins, vegetable fibres, beet pulp, minerals, soya oil, yeasts and parts thereof, fish oil, fructo-oligo-saccharides, psyllium husks and seeds, hydrolysed crustaceans (source of glucosamine), marigold extract (source of lutein), hydrolysed cartilage (source of chondroitin). Vitamin A: 22500 IU, Vitamin D3: 900 IU, Vitamin C: 290 IU, E1 (Iron): 34 mg, E2 (Iodine): 3.4 mg, E4 (Copper): 10 mg, E5 (Manganese): 44 mg, E6 (Zinc): 131 mg, E8 (Selenium): 0.05 mg - Technological additives: Clinoptilolite of sedimentary origin: 5 g - Sensory additives: Tea extract (source of polyphenols): 0.6 g - Zootechnical additives: Ammonium chloride: 5 g - Preservatives - Antioxidants. *L.I.P. : protein selected for its very high assimilation.
**Royal Canin® Ageing 12+ dry**	Maize, vegetable protein isolate*, maize flour, dehydrated poultry protein, wheat, maize gluten, animal fats, vegetable fibres, hydrolysed animal proteins, chicory pulp, minerals, fish oil, soya oil, yeasts and parts thereof, tomato (source of lycopene), fructooligo- saccharides, psyllium husks and seeds, hydrolysed yeast (source of mannan-oligo-saccharides), hydrolysed crustaceans (source of glucosamine), borage oil, marigold extract (source of lutein), hydrolysed cartilage (source of chondroitin). Vitamin A: 19000 IU, Vitamin D3: 700 IU, E1 (Iron): 40 mg, E2 (Iodine): 4 mg, E4 (Copper): 12 mg, E5 (Manganese): 52 mg, E6 (Zinc): 154 mg, E8 (Selenium): 0.07 mg - Sensory additives: Tea extract (source of polyphenols): 0.6 g - Preservatives - Antioxidants. *L.I.P. : protein selected for its very high assimilation.

 https://doi.org/10.3390/ani16010045

Table S2. Initial cat body condition scores (BCS) submitted by owners

	BCS category	n
	2	1
	3	3
	4	18
	5	42
	6	27
	7	21
	8	7
**Total**	-	119
**Mean**	5.5	-

BCS: body condition score. Owners submitted an initial subjective BCS of their cat, using the Royal Canin Body Condition Score chart, based on the published 9-point scale of Laflamme 1997.

Table S3. Questionnaire used by owners following each dietary exposure

Senior Cats Questionnaire

Questionnaire to be sent out on all 18 days of diet exposure

Wednesday 27th November - Day 3 of Diet 2

1. Your name

2. Your cat's name

3. REF number

4. Diet type

Dry	
Wet	
Dry & Wet	

5. How many grams of food has <cat name> eaten today?

If on Dry & Wet diet

6. How much dry food did <cat name> eat?

If on Dry & Wet diet

7. How much wet food did <cat name> eat?

8. How many feeds has <cat name> had today?

9. How long did <cat name> spend at the feeder?

10. How many millilitres of water has <cat name> drunk today?

Thank you. Please record the details of each of the <number from Q8> feeds on the printed table provided to you, making sure to note down the day these feeds occurred on.

Press "submit" to send us your response for this day.

 https://doi.org/10.3390/ani16010045

Please update the feed information on this table **at the end of each day**.

For the days you are on either Wet only or Dry only diet, leave the other column blank. If you are on the Wet & Dry diet and your cat does not eat any of either type, put 0g in that column.

**Date**	**Time**	**Length**	**Dry consumed**	**Wet consumed**
27/11/19	09:58	03:42 mins	25g	14g
“	11:14	52 s	5g	2g

Table S4. Analysis of feeding frequency (number of meals) in a 24-hour period, according to dietary regimen.

Data analyzed	Dietary regimen	24-hour feeding frequency mean estimate	95% CI
**2-day exposure**	Dry	6.0 ^{ab}	5.2 – 6.7
	Wet	6.9^{a}	5.9 – 8.0
	Wet/dry	7.2^{b}	6.3 – 8.2
**6-day analysis**	Dry	6.0^{cd}	5.3 – 6.7
	Wet	7.1^{c}	6.1 – 8.0
	Wet/dry	7.3^{d}	6.5 – 8.2
**Wet/dry mix**	Dry	4.5*	4.0 – 5.0
	Wet	5.6*	4.9 – 6.4

^{a} differences between values of same letter are statistically significant ($p < 0.05$).

^{bcd} differences between values of same letter are statistically significant ($p < 0.001$).

* statistical analysis not performed

Table S5. Analysis of caloric intake in a 24-hour period, according to dietary regimen.

Data analyzed	Dietary regimen	24-hour energy intake mean estimate (kcal)	95% CI
**2-day exposure**	Dry	262.6[a]	218.2 – 307.0
	Wet	138.1[ab]	119.2 – 157.1
	Wet/dry	222.6[b]	189.9 – 255.4
**6-day analysis**	Dry	268.3[ce]	227.0 – 309.6
	Wet	148.3[cd]	130.2 – 166.3
	Wet/dry	224.5[de]	197.2 – 251.8
**Wet/dry mix**	Dry	263.7[f]	217.3 – 310.2
	Wet	182.6[f]	157.1 – 208.0

[abcdf] differences between values of same letter are statistically significant ($p < 0.001$).

[e] differences between values of same letter are statistically significant ($p < 0.05$).

Table S6. Analysis of voluntary water intake (water drunk in g) and total water intake (voluntary plus dietary moisture in g) in a 24-hour period, according to dietary regimen.

Data analyzed	Dietary regimen	24-hour mean estimate (g)	95% CI
**Voluntary water intake**	Dry	62.1[a]	50.9 – 73.2
	Wet	43.3[a]	32.2 – 54.5
	Wet/dry	52.4[a]	41.3 – 63.6
**Total water intake**	Dry	65.9[b]	50.7 – 81.0
	Wet	179.3[b]	164.2 – 194.3
	Wet/dry	139.2[b]	124.1 – 154.4

[ab] differences between values of same letter are statistically significant ($p < 0.001$). Data analyzed across 6-day feeding period.

Table S7. Effect of gender on 24-hour feeding frequency, by dietary regimen

Dietary regimen	Gender	24-hour feeding frequency mean estimate	95% CI	*p* value
**Dry**	Male	6.0	5.0 – 7.1	0.96
	Female	6.0	5.0 – 6.9	
**Wet**	Male	6.8	5.4 – 8.2	0.49
	Female	7.4	6.1 – 8.6	
**Wet/dry**	Male	7.0	5.7 – 8.3	0.46
	Female	7.6	6.4 – 8.8	

Data analyzed across 6-day feeding period by linear fixed effects model fitted with number of meals as response variable, gender as a fixed effect and individual cat as a random effect.

Table S8. Effect of age category on 24-hour feeding frequency, by dietary regimen

Dietary regimen	Age category (years)	24-hour feeding frequency mean estimate	95% CI	*p* value
**Dry**	7-11	6.2	5.3 – 7.2	0.44
	12+	5.7	4.7 – 6.8	
**Wet**	7-11	6.7	5.4 – 8.0	0.29
	12+	7.6	6.2 – 8.9	
**Wet/dry**	7-11	7.2	5.9 – 8.4	0.64
	12+	7.5	6.3 – 8.8	

Data analyzed across 6-day feeding period by linear fixed effects model fitted with number of meals as response variable, age category as a fixed effect and individual cat as a random effect.

Table S9. Effect of gender on 24-hour caloric intake, voluntary water intake and total water intake.

Data analyzed	Gender	24-hour mean estimate	95% CI	*p* value
**Caloric intake (kcal)**	Male	152.7	126.3 – 179.0	0.71
	Female	146.8	123.0 – 170.6	
**Voluntary water intake (g)**	Male	51.7	34.6 – 68.8	0.89
	Female	53.1	37.7 – 68.5	
**Total water intake (g)**	Male	131.2	109.2 – 153.2	0.83
	Female	128.4	108.6 – 148.2	

Data analyzed across 6-day feeding period by linear fixed effects model. For analysis of caloric intake, the model was fitted with caloric intake and gender as fixed effects and dietary format crossed with individual cat as random effects. For analysis of water intake, the model was fitted with water intake as the response variable, gender as a fixed effect and individual cat a random effect.

Table S10. Effect of age category on 24-hour caloric intake, voluntary water intake and total water intake

**Data analyzed**	**Age category (years)**	**24-hour mean estimate**	**95% CI**	***p* value**
**Caloric intake (kcal)**	7-11	151.4	127.0 – 175.7	0.80
	12+	147.3	121.7 – 173.0	
**Voluntary water intake (g)**	7-11	59.2	43.5 – 74.9	0.16
	12+	45.0	28.6 – 61.5	
**Total water intake (g)**	7-11	140.4	120.3 – 160.5	0.08
	12+	117.9	96.8 – 139.0	

Data analyzed across 6-day feeding period by linear fixed effects model. For analysis of caloric intake, the model was fitted with caloric intake and age category as fixed effects and dietary format crossed with individual cat as random effects. For analysis of water intake, the model was fitted with water intake as the response variable, age category as a fixed effect and individual cat a random effect.

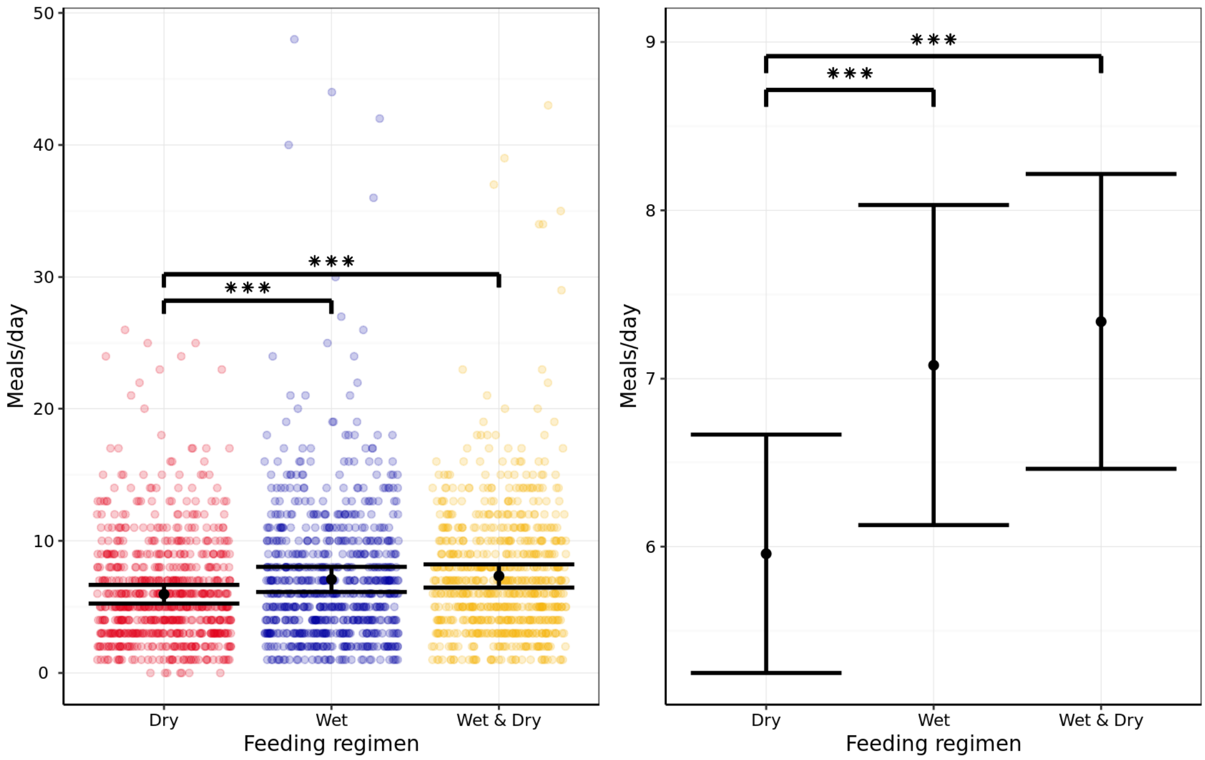

Figure S1. 24-hour frequency of feeding (meals per day) in pet aging cats (n=134) fed all-dry, all-wet or mixed wet/dry regimens. Graphs show (a) individual data points and (b) group means with error bars representing lower and upper 95% CI. Data analyzed from six-day feeding. *** denotes difference between regimens is significant at $p < 0.001$.

 https://doi.org/10.3390/ani16010045

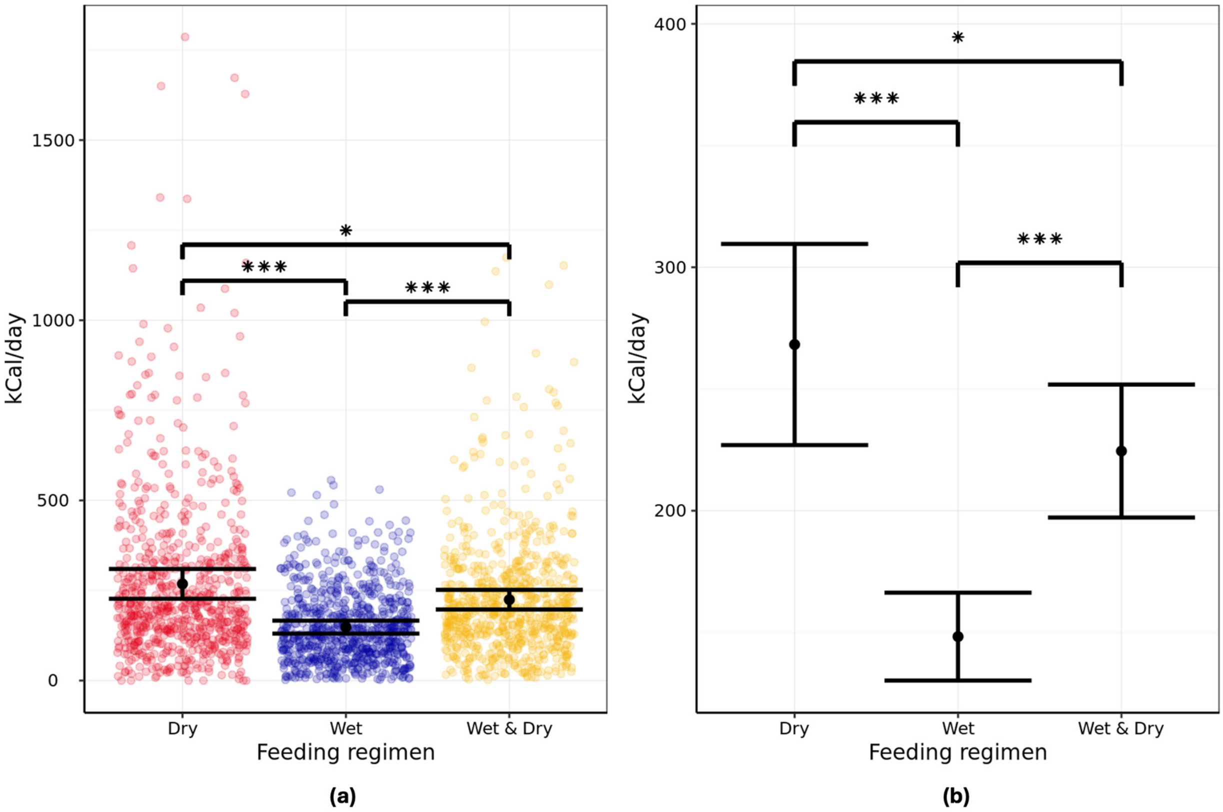

Figure S2. 24-hour energy intake (kcal/day) in pet aging cats (n=134) fed all-dry, all-wet or mixed wet/dry regimens. Graphs show (a) individual data points and (b) group means with error bars representing lower and upper 95% CI. Data analyzed from six-day feeding. * denotes difference between regimens is significant at $p < 0.05$. *** denotes difference between regimens is significant at $p < 0.001$.
